# Multifactorial Etiology Pulmonary Hypertension in a Patient with Sarcoidosis

**DOI:** 10.1155/2016/2481369

**Published:** 2016-10-09

**Authors:** Barreto Ana Terra Fonseca, Barreto Lucas Vinícius da Fonseca, Cavalcante Felipe Naze Rodrigues, Oliveira Joselina Luzia Menezes, Almeida-Santos Marcos Antônio, Garcez Juliane Dantas Seabra, Barreto-Filho José Augusto Soares, Sousa Antônio Carlos Sobral

**Affiliations:** ^1^Departamento de Medicina, Universidade Federal de Sergipe (UFS), São Cristóvão, SE, Brazil; ^2^Departamento de Medicina, Universidade Tiradentes, Aracaju, SE, Brazil; ^3^Centro de Ensino e Pesquisa da Fundação Lucas, Aracaju, SE, Brazil; ^4^Núcleo de Pós-Graduação em Medicina da UFS, São Cristóvão, SE, Brazil; ^5^The American College of Cardiology, New York, NY, USA

## Abstract

Differential diagnosis between pre- and postcapillary pulmonary hypertension (PH) in patients with diastolic heart failure (DHF) is a challenge in clinical practice. The presence of PH is implicated in worse prognosis in patients with this disease. This case report approaches the process of investigation of pulmonary hypertension in adult patient with DHF, double mitral lesion, and sarcoidosis with poor clinical outcome.

## 1. Introduction

Pulmonary hypertension (PH) is characterized by pulmonary artery medium pressure (PAPm) ≥25 mmHg at rest, measured by right heart catheterization. It could be due to a primary increase in the pulmonary arterial system (PAH) or, when secondary, to the increase in the venous system pressure as well within the pulmonary capillary (pulmonary venous hypertension). Also, it can happen in association with a series of clinical conditions [1]. In clinical practice, however, its interpretation, etiologic diagnosis, and therapeutic approach are still a challenge for clinicians.

The pathogenesis of PH is complex and, in most cases, multifactorial. Pulmonary arterial hypertension (PH group 1) is a proliferative vasculopathy of pulmonary arteriolar musculature. On the other hand, the pathophysiology of PH secondary to left heart failure (HF), PH postcapillary or group 2, is less understood. However, it is established that there may be an overlap of these PH etiologies since vascular remodeling and increased pulmonary vascular resistance are common features in both groups. For this group, more studies to evaluate the real benefit of therapy for PH are necessary [2].

Among the most emblematic diagnostic challenges, we could cite patients in whom left HF does not explain the degree of PH or when there is a second potential etiology.

In sarcoidosis (PH group 5), pathological mechanisms of PH are also multiple and complex: pulmonary fibrosis and hypoxemia, granulomatous involvement of arterioles or pulmonary veins, and compression of the proximal pulmonary arteries by hilar lymph nodes [3].

We describe a case report of a dialytic patient, with association of pulmonary sarcoidosis, HF with preserved ejection fraction (HFpEF), PH, and mitral valve disease refractory to optimal medical treatment.

## 2. Case Report

A male patient, 53 years old, was admitted to the internal medicine ward of the University Hospital of the Federal University of Sergipe on 31/3/15 with dyspnea at rest, orthopnea, nocturnal paroxystic dyspnea, and edema of the lower limbs during one month. He reported dyspnea related to moderate to intense efforts, starting 18 years earlier (when pulmonary sarcoidosis was suspected and confirmed through transbronchial lung biopsy). In the last 4 years, he mentioned mild effort dyspnea/leg edema. The echocardiography exam presented moderate mitral stenosis with severe calcification of the valve and subvalve apparatus, in spite of the lack of a rheumatic disease history. Among the comorbidities, he presented signs and symptoms of severe chronic renal failure for the earlier six years (actually, he was under dialysis for about 4.5 years) and epilepsy (about 15 years). He was a former smoker (15 packs/year) and referred to previous exposure to dust in work place.

He took regularly metoprolol 50 mg/day, Amlodipine 2.5 mg/day, Sildenafil 20 mg q8 hr, hemodialysis q2 days, prednisone 10 mg/day, Sevelamer 800 mg q8 hr, Phenobarbital 100 mg/day, and the folic acid/calcitriol. He reported episodes of symptomatic hypotension, a fact that do not allow drug optimization of heart failure (HF) and motivated hospitalization for clinical compensation.

On physical examination, his skin was pale, albeit hydrated. In the cardiac auscultation, there was a regular rhythm, presence of premature beats, grade III mitral, and tricuspid systolic murmur, accentuated second sound. The heart rate was 92 beats per minute and the blood pressure was 92 × 58 mmHg. We also noticed jugular venous distension, even while standing. In the lung auscultation, murmurs were present in both sides, but markedly decreased at right lower lobe. The respiratory rate was 28 breaths per minute. The abdomen was flat, and we could touch the inferior boarder of the liver at four centimeters from the costal margin (painful on palpation). There was evidence of edema within the abdominal wall. The skin was hot and dry, there was ankle pitting, and we could classify the degree of edema as 3+ (maximum = 4) in the tibiae. The peripheral pulse was present and symmetric.

**Figure 1:**
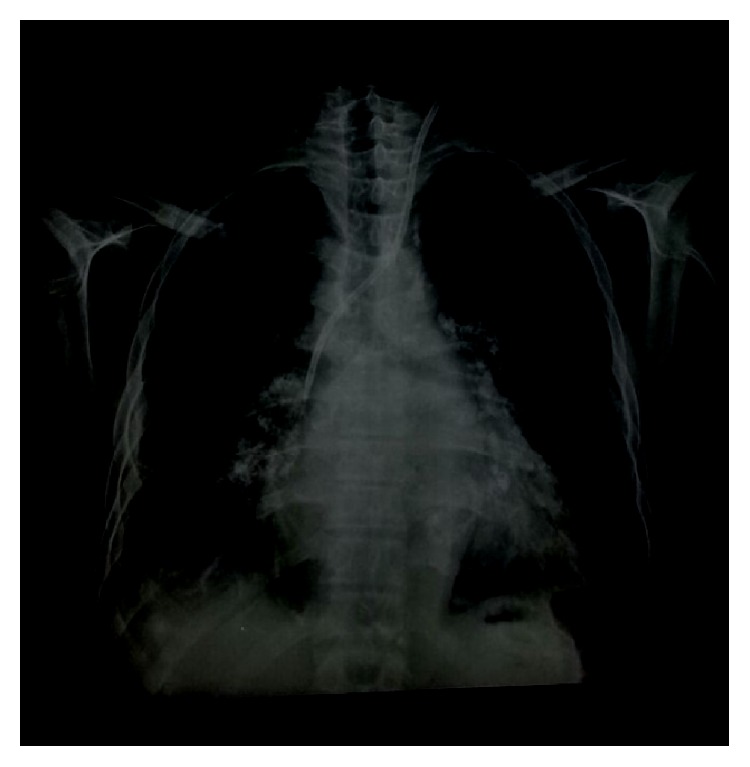
Increased cardiac area, middle arch rectification, left atrial increase, bilateral hilar lymphadenopathy, and calcified and Kerley B lines.

The chest radiography (Figure 1) showed an increased cardiac area, middle arch rectification, left atrial increase, bilateral calcified hilar lymphadenopathy, and Kerley B lines. The electrocardiogram presented sinus rhythm with heart rate of 92 bpm, right axis deviation, divisional posterior inferior blocking, and increased P (biatrial overload) and T waves (secondary changes in ventricular repolarization). The evolutionary findings of EchoDopplercardiograms (ED) transthoracic revealed progression of mitral stenosis (mitral valve opening evolved from 1.35 cm^2^ to 1 cm^2^), extensive calcification of the mitral valve (score of Wilkin's 15 points), moderate aortic and tricuspid insufficiency, and normofunctioning pulmonary valve. The systolic pulmonary artery pressure (PASP) worsened (48 mmHg to 79 mmHg) and micronodular hyperechoic images were noted diffusely in left ventricle myocardium. Comparative description of diameters and ejection fraction are shown in Table 1.

Hemodialysis was instituted daily in order to improve clinical status. The doses of metoprolol (150 mg/day) and prednisone (40 mg/day) were increased. However, the approaches adopted have not accomplished any success.

The right heart catheterization was performed for the investigation of pulmonary arterial hypertension with nitroprusside 200 *μ*g/mL at a dose of 2.8 mcg/kg/min for 1 h. The following pressures were obtained: (a) before vasodilator: pulmonary artery pressure, 76/30 mmHg (mean 51) and Aorta, 96/76 mmHg (mean 85); (b) after vasodilator: pulmonary artery, 65/31 mmHg (mean 49) and Aorta, 104/53 mmHg (mean 70 mmHg). The end-diastolic pressure of the left ventricle was 20 mmHg and coronary angiography revealed only mild diffuse parietal irregularities.

A few days later, the patient developed drowsiness, tachypnea, use of accessory respiratory muscles, 94% of arterial oxygen saturation on supplemental oxygen 3 L/min, blood pressure 70 × 50 mmHg, heart rate 112 beats per minute, worsening of mitral systolic murmur (grade IV) and mitral diastolic murmur, cold and clammy skin, and weak pulse. He was transferred to the intensive care unit to receive hemodynamic support with vasoactive drugs, but he evolved with refractory shock and death.

## 3. Discussion

The PH is a common complication of left HF; usually its manifestation often overlaps with those of underlying condition and implicates more severe clinical presentation. In clinical practice, the diagnosis is usually done indirectly, through transthoracic ED. However, their interpretation, main diagnosis, and therapeutic approach still are a challenge for clinician [4].

The comparative analysis of ED showed unfavorable evolution of mitral stenosis, valve area reduction, left atrial enlargement, worsening of PSAP, and right chambers enlargement. These data corroborate the etiology of postcapillary PH in this patient (group 2 PH, associated with left heart disease) [5].

The postcapillary PH is more common in HFpEF which consists of abnormality in active relaxation and active stiffness (diastolic dysfunction) with preserved left ventricular ejection fraction. When HFpEF occurs, it results in more severe symptoms and worsening of exercise tolerance, hallmark of the patient in question [4, 6].

As there was not found satisfactory reduction in PAPm under vasodilator testing (pulmonary reactivity criteria: fall PAPm to <49 mmHg, reduction of at least 10 mmHg, or maintenance/increase in cardiac output) and the estimated pulmonary capillary wedge pressure was 20 mmHg, it infers fix PH with postcapillary component probably secondary to valve heart disease. The differential diagnosis between PH modalities before and after capillary in patients with HFpEF is a challenge in daily clinical practice and it entails difficulty on treating this subgroup of patients. However, the literature states that group 2 PH patients can have precapillary component, featuring a mixed PH [2]. Thus, it was decided to maintain the treatment with phosphodiesterase 5 inhibitors even after the above examinations do not corroborate primary etiology. However, more studies are needed to evaluate the benefit of therapy for PH in this group.

The diagnosis of PH, by itself, cannot exclude the possibility of it being due to mitral valve disease in this patient. However, the prescription of sildenafil started long before the clinical presentation of the valve disease and, at that early phase, the treatment improved the patient condition. Notwithstanding, we must acknowledge that sildenafil could potentially contribute to the clinical deterioration of the patient. Indeed, due to an increase of symptoms a few weeks before the fatal event, the use of sildenafil was put to a halt.

Myocardial infiltration, sarcoidosis characteristic, viewed at ED probably did not contribute to the clinical presentation, as usually it manifests itself with restrictive cardiomyopathy, ventricular aneurysm, pericardial disease, infiltrative valve dysfunction, ventricular tachycardia, and supraventricular arrhythmias [3].

In this case the surgical approach of mitral valve was not possible. The patient did not have favorable valve anatomical conditions to mitral stenosis percutaneous repair and also had high surgical risk (severe pulmonary hypertension and disorders of right ventricle and kidneys) to mitral valve replacement. There was no possibility to carry out more radical therapies like cardiac transplantation because of severe fixed pulmonary hypertension and atrial septostomy because of high mortality inherent to it [7].

It is very difficult to determine the time when HF patient care should be palliative. Anguita and Ojeda [8] defined a few points to guidance in making this decision: class functional III-IV despite optimal treatment, absence of triggering factors or advanced treatable comorbidities that limit survival, hypotension, renal failure, and frequent decompensation despite maximum tolerated treatment. All these prognostic factors were presented in this case and it was featured as terminal HF.

## Figures and Tables

**Table 1:** Transthoracic echocardiograms.

	09/04/15 (admission)	07/11/13
Aorta/left atrium (cm)	3.1/6.1 (volume = 78 mL/m^2^)	3.2/5.5
Right chambers	Enlarged	Normal
Left ventricle (diastole/systole) (cm)	3.8/2.2	4.1/2.3
Ejection fraction (Simpson)	74%	76%
